# Reproductive and obstetric outcomes following frozen embryo transfer: letrozole combined with human menopausal gonadotropin versus hormone replacement cycle

**DOI:** 10.3389/fendo.2026.1698208

**Published:** 2026-01-29

**Authors:** Hui-Juan Guan, Huai-Yun Tang, Hui Li, Jia-Hui Zhao, Jia Song, Li-Sha Tang, Yan-Lan Hao

**Affiliations:** 1 Clinical Center of Reproductive Medicine, Lianyungang Maternal and Child Health Hospital, Lianyungang, Jiangsu, China; 2 Department of Obstetrics, Lianyungang Maternal and Child Health Hospital, Lianyungang, Jiangsu, China

**Keywords:** frozen-thawed embryo transfer, hormone replacement therapy, obstetric outcome, ovulation induction, reproductive

## Abstract

**Background:**

This study aimed to compare reproductive and obstetric outcomes between frozen embryo transfer (FET) cycles using letrozole combined with human menopausal gonadotropin (HMG) for ovulation induction (OI) and hormone replacement therapy (HRT) for endometrial preparation.

**Methods:**

A retrospective cohort study was conducted on 1,880 FET cycles from 2016 to 2024. Cycles were stratified into the OI group (*n* = 902) and HRT group (*n* = 978) based on the endometrial preparation protocol. The primary outcome was live birth rate (LBR). Secondary outcomes included clinical pregnancy rate (CPR) and obstetric complications. Exploratory subgroup analyses were performed based on ovulatory status (normal ovulation vs. ovulation disorders) and age (<35 years vs. ≥35 years).

**Results:**

After adjustment for confounders, no statistically significant difference was observed in the primary outcome of LBR between the OI and HRT groups (OR = 1.145, 95% CI: 0.932–1.407; *P* = 0.198). Similarly, there was no significant difference in the secondary outcome of CPR (OR = 1.149, 95% CI: 0.944–1.398; *P* = 0.167). Analysis of other secondary outcomes revealed that the OI protocol was associated with a lower risk of cesarean section (OR = 0.619, 95% CI: 0.432–0.887; *P* = 0.009) and gestational diabetes mellitus (GDM) (OR = 0.339, 95% CI: 0.117–0.981; *P* = 0.046). Exploratory subgroup analyses suggested potential variations: In women with ovulation disorders, OI was associated with a higher CPR (OR = 1.624, 95% CI: 1.081–2.440; *P* = 0.020) and a lower preterm birth rate (OR = 0.682, 95% CI: 0.408–0.562; *P* = 0.023). In women ≥35 years, OI was associated with a markedly lower risk of GDM (OR = 0.038, 95% CI: 0.002–0.707; *P* = 0.028) and a non-significant trend toward higher LBR (OR = 1.521, 95% CI: 0.967–2.393; *P* = 0.07). In women <35 years, the OI cycle was associated with a lower cesarean section rate (OR = 641, 95% CI: 0.426–0.996; *P* = 0.034).

**Conclusions:**

Adjusted analysis revealed comparable LBR between the OI and HRT protocols. The OI protocol was associated with a lower risk of cesarean section and GDM in the overall population, with exploratory subgroup analyses suggesting potential differential effects in specific patient groups. These findings warrant prospective validation.

## Introduction

Over the past decade, frozen embryo transfer (FET) has become increasingly common, accounting for 30%–40% of all embryo transfers globally (1, 2). By preserving surplus embryos generated during controlled ovarian hyperstimulation (COH) and enabling transfer in a more physiologically synchronized uterine environment, FET may enhance embryo implantation rates (3). However, the optimal endometrial preparation protocol for maximizing pregnancy and obstetric outcomes remains a topic of debate.

Currently, three main endometrial preparation protocols are used for FET: natural cycles, hormone replacement therapy (HRT) cycles, and mild ovulation induction (OI) cycles. The choice of protocol typically depends on physician preference and the patient’s ovulatory function (4).

Previous retrospective studies suggest that OI protocols may yield higher live birth rates (LBR) than HRT in women with polycystic ovary syndrome (PCOS) and reduce hypertensive disorders in pregnancy (5–8). A recent meta-analysis further confirmed that OI increases LBR and decreases the risks of miscarriage, preeclampsia, and preterm birth in PCOS patients (9). However, one randomized controlled clinical trial (RCT) found no significant differences in cardiopulmonary outcomes between the two protocols (10).

Letrozole (LE) is a commonly used ovulation inducer in OI protocols. While LE combined with HMG is increasingly applied in FET, data on its pregnancy and perinatal outcomes remain limited. This study aimed to address key questions: does LE+HMG-based endometrial preparation affect reproductive outcomes? Do outcomes differ by population subgroups (e.g., ovulatory status, age)? To this end, we conducted a retrospective analysis of 1,880 FET cycles to explore these questions.

## Materials and methods

### Study design and ethics

This retrospective cohort study included FET cycles conducted at the Lianyungang Maternal and Child Health Hospital from January 2016 to December 2024. This study was approved by the Ethics Committee of the hospital’s Research Ethics Board (Approval No.: LYG-MER2021022). Given its retrospective observational design and the fact that all analyses were performed using anonymized data, the committee waived the requirement for obtaining written informed consent from the patients.

### Patients

#### Patient selection

Included patients underwent their first FET cycle, with endometrial preparation performed using either OI (LE+HMG) or HRT. Exclusion criteria were as follows: (1) History of recurrent spontaneous abortion (≥ 3 consecutive miscarriages); (2) Uterine abnormalities (e.g., bicornuate uterus, moderate-to-severe intrauterine adhesions, uterine fibroids >5 cm or fibroids causing uterine cavity distortion); (3) Severe endometriosis or untreated hydrosalpinx; (4) Pre-existing hypertension or diabetes mellitus; (5) Need for preimplantation genetic testing (PGT) of embryos.

The selection of the endometrial preparation protocols is determined by the patient’s previous ovarian stimulation cycle and personal schedule. HRT cycles with gonadotropin-releasing hormone (GnRH) agonists were also excluded, as they are rarely used in our center (primarily reserved for patients with recurrent implantation failure, adenomyosis, or severe endometriosis). Of the initial 3,621 FET cycles screened, 1,880 met the inclusion criteria with complete data (no missing values for key variables).

#### Embryo culture, cryopreservation, and thawing

All patients first underwent controlled ovarian hyperstimulation (COH). Oocyte retrieval was performed 34–36h after administration of the ovulation trigger. Embryos were cultured at 37 °C under a gas mixture of 5% CO_2_, 5% O_2_, and 90% N_2_.

Cleavage-stage embryos were graded by morphological criteria (11), with high-quality embryos defined as Grades I–II. Blastocysts were scored using the Gardner system (12), with good-quality blastocysts ≥3BB (AA, AB, BA, BB). Vitrification was performed using the Kitazato open system with DMSO-EG as a cryoprotectant. Thawing used the Kitazato thawing kit. Embryos were considered viable if ≥50% of blastomeres survived (cleavage stage) or the blastocyst cavity re-expanded within 2h (blastocysts). The embryo survival rate at our center was approximately 90%.

#### Endometrial preparation

All patients underwent baseline assessment on menstrual day 3: serum follicle-stimulating hormone, luteinizing hormone, and estradiol (E_2_); transvaginal ultrasound (performed by standardized, trained physicians). Endometrial preparation started if there are no follicles >10 mm, endometrial thickness ≤8 mm, and E_2_<80pg/ml.

#### OI cycle

Medication administration: From menstrual cycle days 3 to 5, patients received LE (Jiangsu Hengrui Pharmaceutical Co., Ltd., Lianyungang City, Jiangsu Province, China) at a daily dose of 5 mg for 5 consecutive days, combined with HMG (Livzon Pharmaceutical Co., Ltd., Lianyungang City, Jiangsu Province, China) at 75 IU administered every other day.

Monitoring: Transvaginal ultrasound was performed 1 week after the start of medication to assess follicle diameter and endometrial thickness/type (classified per the Gonen system: Type A, B, or C) (13). Medication was adjusted based on follicle and endometrial parameters. If no follicle ≥10 mm was observed, HMG was increased to 75 IU daily. Oral estradiol was added if needed to increase endometrial thickness:if the diameter of the dominant follicle reached ≥14 mm, but the endometrial thickness remained <7 mm, oral estradiol at a daily dose of 2–4 mg was administered to promote endometrial growth. Following supplementation, ultrasound monitoring was repeated every 2–3 days, and embryo transfer proceeded when the endometrial thickness reached ≥7 mm. If the endometrial thickness remained <7 mm before the scheduled transfer, the cycle was canceled.

Trigger and transfer: 8000 IU human chorionic gonadotropin (hCG, Livzon) was administered when the dominant follicle reached ≥18 mm. Ovulation was confirmed by ultrasound 36h later, with transfer timing based on ovulation.

Embryo selection: Blastocysts were prioritized. Single blastocyst transfer was standard; a maximum of two embryos can be transferred. For patients with a scarred uterus, only a single embryo is permitted for transfer, whether at the cleavage stage or blastocyst stage.

Luteal support: 200 mg progesterone vaginal suppositories (Besins Healthcare, Lianyungang City, Jiangsu Province, China) twice daily. Cleavage embryos were transferred on day 3 of progesterone and blastocysts on day 5. Support continued for 14 days post-transfer; if pregnancy was confirmed and embryonic development proceeds normally, luteal phase support is continued, typically until approximately 8–10 weeks of gestation. By this stage, placental function has become sufficiently established to produce adequate progesterone for maintaining the pregnancy independently. The medication is then gradually tapered and discontinued over a period of 1–2 weeks.

#### HRT cycle

Medication administration: From menstrual cycle day 3, patients received 4 mg oral estradiol daily for the 7 days.

Monitoring: Transvaginal ultrasound was performed after 1 week of estradiol treatment. Endometrial preparation was continued if endometrial thickness reached ≥7 mm, with estradiol administration maintained for a total of 12–14 days. If the endometrial thickness measured less than 7 mm, the dose of estradiol was increased to 6–8 mg per day. After continuing this adjusted regimen for 5–7 days, a follow-up ultrasound examination was performed. This adjustment process could be repeated until the endometrial thickness reached ≥7 mm. If the target thickness was still not achieved after 30 days of estradiol supplementation. There is no unified consensus on the maximum duration of estradiol use. The recommendation of up to 30 days is an empirical suggestion from some experts based on conference discussions. However, since we have already ruled out factors such as intrauterine adhesions, there is no scenario where prolonged use of estradiol is required due to persistently inadequate endometrial thickness. The embryo transfer cycle was canceled.

Embryo selection: Same as OI cycle.

Luteal support: 200 mg progesterone suppositories three times daily plus 10 mg oral dydrogesterone (Abbott Healthcare, Lianyungang City, Jiangsu Province, China) three times daily. Estradiol was continued. Transfer timing and luteal support time matched the OI cycle (cleavage embryos: day 3 of progesterone; blastocysts: day 5).

### Outcome assessment and follow-up visit

Primary outcome: Live birth rate (LBR), defined as the proportion of transferred cycles resulting in at least one live birth delivered at or beyond 28 weeks of gestation.

Secondary outcomes: These included clinical pregnancy rate (CPR, defined as the presence of a gestational sac with fetal heartbeat on transvaginal ultrasound 4–5 weeks after transfer);implantation rate (IR, number of gestational sacs per transferred embryo × 100%), positive hCG rate (proportion of patients with serum hCG >5 IU/L 14 days post-transfer), ectopic pregnancy rate, miscarriage rate (loss of pregnancy before 28 weeks of gestation), preterm birth rate (birth before 37 weeks of gestation), pregnancy-induced hypertension (PIH) rate, and GDM rate.

Data were collected via review of electronic medical records and telephone follow-ups conducted by trained research nurses.

### Statistical analysis

The sample size for this retrospective study was determined by the availability of complete data during the study period. However, the achieved power was calculated based on the observed data. Assuming a baseline LBR of 31.4% in the HRT group (observed in our data), to detect an absolute increase of 6.0% (to 37.4%) in the OI group with 90% power at a two-sided α of 0.05, approximately 400 cycles per group would be required. Our final included sample (902 OI vs. 978 HRT) meets and exceeds this requirement. Data were analyzed using SPSS 22.0 software (IBM, NY, USA). Normally distributed data are presented as mean ± SD; non-normal data as median. Categorical variables are presented as percentages and compared using Pearson’s chi-square or Fisher’s exact test. Logistic regression models were constructed to assess the association between the endometrial preparation protocol (OI vs. HRT) and the primary outcome of LBR, as well as secondary outcomes, while adjusting for prespecified confounders.

Logistic regression was used to calculate adjusted odds ratios (ORs) for all binary outcomes. For outcomes with intermediate incidence [i.e., not rare (<10%) nor very common (>90%)], such as clinical pregnancy and live birth, the OR provides a close approximation to the risk ratio (RR) and is the standard metric reported in observational reproductive medicine studies (13). For rare outcomes, results are interpreted with caution, noting the small number of events.

## Results

### Baseline characteristics

Compared with the HRT group, the OI group had a significantly lower body mass index (BMI) (*P* = 0.001). Additionally, the OI group exhibited a higher proportion of Type A endometrium (per Gonen classification), lower proportions of Type B/C endometrium, greater endometrial thickness, a higher rate of blastocyst transfer (62.97% vs. 38.85%), a higher proportion of high-quality embryos transferred (86.36% vs. 79.55%), and a lower number of embryos transferred per cycle (all *P* < 0.001) (**Table 1**).

**Table 1:** Comparison of baseline characteristics of the population.

Variables	HRT	OI	P-value
No of cases	978	902	**-**
Years			
Women	32 (29, 35)	32 (29, 34)	0.845
Men	32 (29, 36)	32 (30, 35)	0.465
AMH (ng/ml)	3.96 (2.33, 6.44)	4.01 (2.45, 6.30)	0.668
Maternal BMI (kg/m² )	23.4 (21.2, 25.96)	22.9 (20.7, 25.36)	0.001
Infertility diagnosis, n (%)			
Primary	428 (43.76)	368 (40.80)	0.208
secondary	550 (56.23)	534 (59.20)	
History of abortion	222	176	0.091
Duration of infertility (y)	4 (2, 6)	4 (2, 6)	0.161
Infertility etiology, n (%)			
Pelvic factors	568 (58.08)	515 (57.10)	
Endometriosis	20 (2.04)	10 (1.11)	
Ovulatory dysfunction	244 (24.95)	211 (23.39)	0.063
Men	54 (5.52)	80 (8.86)	
Both parties’ factors	81 (8.28)	77 (8.53)	
Unexplained	11 (1.12)	9 (1.00)	
Basic endocrine level			
Basal FSH (uIU/ml)	6.19 (5.08, 7.44)	5.78 (4.76, 6.98)	< 0.001^*^
Basal LH (uIU/ml)	4.68 (3.27, 7.03)	4.32 (3.08, 6.33)	< 0.001^*^
Basal E_2_ (pmol/L)	35 (25, 43)	32 (24, 41)	0.001^*^
Endometrial thickness (mm)	10.60 (9.0, 12.0)	11 (9.0, 12.0)	0.036
Endometrial type, n (%)			
A	118 (12.07)	165 (18.29)	
B	814 (83.23)	715 (79.27)	< 0.001^*^
C	46 (4.70)	22 (2.43)	
Embryos stage, n (%)			
Cleavage embryo	598 (61.15)	334 (37.03)	< 0.001^*^
Blastocyst	380 (38.85)	568 (62.97)	
No. of embryo transferred, n (%)			
Single	561 (57.36)	650 (72.06)	< 0.001^*^
Double	417 (42.64)	252 (27.94)	
Embryo quality, n (%)			
At least one high-quality embryos	778 (79.55)	779 (86.36)	< 0.001^*^
Non-quality embryos	200 (20.45)	123 (13.64)	

OI, Ovulation induction; HRT, Hormone replacement treatment; AMH, Anti-mullerian hormone; BMI, Body mass index; No, number. *P-values < 0.05.

These differences were further evaluated in multivariate models, with the primary focus on the adjusted LBR.

This study employs a retrospective design, and baseline differences exist between the two patient groups, reflecting real-world clinical decision-making patterns. Specifically, compared to the HRT group, patients in the OI group had a lower BMI and more favorable endometrial status, characterized by a higher proportion of Type A endometrium and greater endometrial thickness. Concurrently, the OI group exhibited higher rates of blastocyst transfer and single embryo transfer, yet a lower proportion of morphologically high-quality embryos. All these factors are significant predictors of pregnancy outcomes. To control for these confounders, we systematically adjusted for BMI, endometrial parameters, embryo stage (blastocyst/cleavage), embryo quality score, and the number of embryos transferred in our multivariable logistic regression model.

### Unadjusted outcomes

Univariate analysis revealed that the OI group had significantly higher rates of positive hCG testing, IR, CPR, and LBR, as well as significantly lower rates of cesarean section, PIH, and GDM (all *P* < 0.05). No significant differences were observed between the two groups in miscarriage rate, ectopic pregnancy rate, multiple pregnancy rate, preterm birth rate, or macrosomia rate (**Table 2**).

**Table 2:** Reproductive and obstetric outcomes.

Pregnancy outcome	HRT	OI	P-value
Implantation Rate, n (%)	487 (34.91)	518 (44.89)	< 0.001^*^
Positive hCG rate, n (%)	481 (48.73)	501 (55.54)	0.014^*^
Clinical Pregnancy rate n (%)	426 (43.16)	463 (51.33)	< 0.001^*^
Ectopic pregnancy rate n (%)	5 (1.17)	6 (1.30)	0.869
Miscarriage rate, n (%)	70 (16.43)	56 (15.55)	0.720
Live birth rate, n (%)	310 (31.41)	338 (37.47)	0.006^*^
Multiple pregnancy rate, n (%)	44 (7.98)	37 (6.48)	0.226
Newborn’s sex (Male) n (%)	155 (50.00)	175 (51.78)	0.652
Newborn’s sex (Female) n (%)	155 (50.00)	163 (48.22)	
Cesarean section, n (%)	234 (75.48)	221 (65.38)	0.005^*^
preterm birth, n (%)	32 (13.01)	41 (15.13)	0.667
Low birth weight, n (%)	22 (7.10)	23 (6.80)	0.884
Macrosomia, n (%)	36 (11.61)	36 (10.65)	0.368
Pregnancy-induced hypertension, n(%)	14 (4.52)	4 (1.18)	0.010^*^
Gestational diabetes, n(%)	15 (4.84)	5 (1.48)	0.014^*^

OI, Ovulation induction; HRT, Hormone replacement treatment. *P-values < 0.05.

### Adjusted outcomes

After adjusting for confounders (age, infertility duration, abortion history, BMI, AMH, infertility etiology, endometrial parameters, and embryo characteristics), logistic regression analysis was performed with the HRT group as the reference. Regarding the primary outcome, no significant difference was observed in LBR between the two groups (OR = 1.145, 95% CI: 0.932–1.407; *P* = 0.198). Similarly, the secondary outcome of CPR did not differ significantly (OR = 1.149, 95% CI: 0.944–1.398; *P* = 0.167). However, analysis of other secondary obstetric outcomes revealed that the OI protocol was associated with a significantly lower risk of cesarean delivery (OR = 0.619, 95% CI: 0.432–0.887; *P* = 0.009) and GDM (OR = 0.339, 95% CI: 0.117–0.981; *P* = 0.046). (**Table 3**).

**Table 3:** Logistic regression of pregnancy and obstetric outcomes.

outcomes	Odds ratio	95% confidence interval	P-value
Clinical pregnancy rate	1.149	0.944-1.398	0.167
**Live birth rate**	**1.145**	**0.932-1.407**	**0.198**
Miscarriage rate	0.926	0.637-1.345	0.686
Ectopic pregnancy rate	1.593	0.516-4.916	0.418
Cesarean section	0.619	0.432-0.887	0.009*
preterm birth	1.601	0.713-3.592	0.254
Low birth weight	0.489	0.096-2.480	0.388
Pregnancy-induced hypertension	0.401	0.123-1.304	0.129
Gestational Diabetes	0.339	0.117-0.981	0.046^*^

The primary outcome is highlighted in bold. The reference group was the HRT group. An OR > 1 indicates a higher risk in the OI group, while an OR < 1 indicates a lower risk in the OI group. Adjusted for the women age, duration of infertility, history of abortion, BMI, AMH, infertility etiology, the endometrial thickness and type, the number of embryos transferred, embryo stage and quality. OR, odds ratio; CI, confidence interval; *P-values < 0.05.

We conducted exploratory subgroup analyses to assess potential differences in outcomes based on ovulatory status and maternal age.

### Subgroup analysis by ovulatory status

#### Women with normal ovulation

The OI group had significantly higher IR (*P* < 0.05) and CPR (*P* = 0.041), as well as significantly lower rates of cesarean section (*P* = 0.005), PIH (*P* = 0.009), and GDM (*P* = 0.048) compared to the HRT group. Adjusted analysis confirmed that OI was independently associated with a lower cesarean section rate (OR = 0.514, 95% CI: 0.332–0.796; *P* = 0.003) (**Tables 4**, **5**).

**Table 4:** Subgroup analysis of reproductive and obstetric outcomes.

Pregnancy outcome	normal ovulation		P value	abnormal ovulation		P value
	HRT	OI		HRT	OI	
Implantation Rate,n(%)	366 (35.26)	464 (50.99)	< 0.001^*^	116 (32.49)	119 (48.77)	< 0.001^*^
Positive hCG rate n(%)	354 (48.83)	384 (53.56)	0.072	127 (50.20)	117 (63.24)	0.007^*^
Clinical Pregnancy rate, n(%)	322 (44.41)	357 (49.79)	0.041^*^	104 (41.11)	106 (57.30)	0.001^*^
Ectopic pregnancy rate, n(%)	4 (1.24)	6 (1.68)	0.672	2 (1.96)	2 (1.92)	0.985
Miscarriage rate n(%)	44 (13.66)	36 (10.08)	0.148	15 (14.42)	18 (16.98)	0.611
Live birth rate, n(%)	231 (31.86)	260 (36.26)	0.078	79 (31.23)	78 (42.16)	0.018^*^
Multiple pregnancy rate, n(%)	55 (7.59)	54 (7.53)	0.969	12 (11.54)	13 (12.26)	0.871
Cesarean section, n(%)	180 (77.92)	173 (66.54)	0.005^*^	54 (68.35)	48 (61.54)	0.371
preterm birth, n(%)	28 (12.12)	34 (13.08)	0.750	15 (18.99)	9 (11.54)	0.195
Lowbirth weight, n(%)	16 (6.93)	19 (7.31)	0.587	6 (7.59)	4 (5.13)	0.527
Macrosomia, n(%)	33 (14.29)	25 (9.62)	0.110	11 (13.92)	11 (14.10)	0.974
Pregnancy-induced hypertension, n(%)	12 (5.19)	3 (1.15)	0.009^*^	2 (2.53)	2 (2.53)	0.947
Gestational Diabetes, n(%)	12 (5.19)	5 (1.92)	0.048^*^	3 (3.8)	NA	NA

OI, Ovulation induction; HRT, Hormone replacement treatment. NA: Due to an insufficient number of events, no meaningful statistical analysis was performed. *P < 0.05. This exploratory subgroup analysis was performed without adjustment for multiple comparisons and should therefore be interpreted with caution.

**Table 5:** Logistic regression of pregnancy and obstetric outcomes for patients with normal and abnormal ovulation respectively.

Outcomes	normal ovulation			abnormal ovulation		
	Adjusted OR	95%CI	p-value	Adjusted OR	95% CI	P-value
Clinical pregnancy	1.048	0.836-1.312	0.686	1.624	1.081-2.440	0.020^*^
Live birth rate	1.093	0.862-1.387	0.463	1.364	0.897-2.072	0.146
Miscarriage rate	0.898	0.583-1.382	0.624	1.194	0.539-2.648	0.662
Ectopic pregnancy	2.320	0.562-9.570	0.244	0.840	0.093-7.607	0.877
Cesarean section	0.514	0.332-0.796	0.003^*^	1.201	0.578-2.499	0.624
preterm birth	0.858	0.481-1.528	0.602	0.682	0.408-1.142	0.023^*^
Lowbirthweight	1.282	0.608-2.706	0.514	NA	NA	NA
Macrosomia	0.538	0.318-1.071	0.082	0.942	0.182-4.876	0.943
Pregnancy-induced hypertension	0.415	0.107-1.610	0.204	1.265	0.473-3.386	0.640
Gestational diabetes	0.400	0.114-1.404	0.153	1.667	0.133-2.093	0.092

Adjusted for women age, BMI, the number of abortion, endometrial preparation methods, endometrial thickness and pattern, the number of embryo transferred and embryos stage. OR, odds ratio; CI, confidence interval; NA:Due to an insufficient number of events, no meaningful statistical analysis was performed. *P < 0.05. This exploratory subgroup analysis was performed without adjustment for multiple comparisons and should therefore be interpreted with caution.

#### Women with ovulation disorders

The OI group had significantly higher IR, positive hCG rate, CPR, and LBR (all *P* < 0.05) compared to the HRT group. Adjusted analysis showed that OI independently improved CPR (OR = 1.624, 95% CI: 1.081–2.440; *P* = 0.020) and a lower preterm birth rate (OR = 0.682, 95% CI: 0.408–0.562; *P* = 0.023) (**Tables 4**, **5**).

### Subgroup analysis by age

#### Women < 35 years

The OI group had significantly higher IR, positive hCG rate, and CPR (all *P* < 0.05) compared to the HRT group. Adjusted analysis revealed that OI was independently associated with a lower cesarean section rate (OR = 0.641, 95% CI: 0.426–0.996; *P* = 0.034) (**Tables 6**, **7**).

**Table 6:** Subgroup analysis of pregnancy outcomes between the two groups.

Pregnancy outcome	<35 years		P value	≥35 years		P value
	HRT	OI		HRT	OI	
Implantation Rate n(%)	382 (37.30)	414 (46.62)	< 0.001^*^	100 (26.95)	98 (36.84)	0.008^*^
Positive hCG rate n(%)	372 (52.69)	401 (58.03)	0.045^*^	109 (40.07)	100 (47.39)	0.107^*^
Clinical Pregnancy rate, n(%)	334 (47.31)	371 (53.69)	0.017^*^	92 (33.82)	92 (43.60)	0.028^*^
Ectopic pregnancy rate, n(%)	5 (1.50)	8 (2.16)	0.516	1 (1.09)	NA	NA
Miscarriage rate, n(%)	48 (14.37)	52 (14.02)	0.893	22 (23.91)	20 (21.74)	0.725
Live birth rate, n(%)	256 (36.26)	276 (39.94)	0.157	54 (19.85)	62 (29.38)	0.015^*^
Multiple pregnancy rate, n(%)	48 (14.37)	43 (11.59)	0.271	8 (8.70)	6 (6.52)	0.578
Cesarean section, n(%)	190 (74.22)	179 (64.86)	0.140	44 (81.48)	42 (67.74)	0.092
preterm birth, n(%)	39 (15.23)	35 (12.68)	0.395	4 (7.41)	8 (12.90)	0.332
Lowbirth weight, n(%)	20 (7.81)	17 (6.16)	0.454	2 (3.70)	6 (9.68)	0.205
Macrosomia, n(%)	36 (14.06)	29 (10.51)	0.211	8 (14.81)	7 (11.29)	0.573
Pregnancy-induced hypertension, n(%)	12 (4.69)	NA	NA	2 (3.70)	2 (3.23)	0.888
Gestational Diabetes, n(%)	8 (3.13)	NA	NA	6 (11.11)	1 (1.61)	0.032^*^

OI, Ovulation induction; HRT, Hormone replacement treatment. NA: Due to an insufficient number of events, no meaningful statistical analysis was performed. *P < 0.05. This exploratory subgroup analysis was performed without adjustment for multiple comparisons and should therefore be interpreted with caution.

**Table 7:** Logistic regression of pregnancy and obstetric outcomes For patients with different years.

outcomes	<35 years		p Value	≥35 years		p Value
	Adjusted OR	95%CI		Adjusted OR	95%CI	
Clinical pregnancy	1.060	0.846-1.330	0.612	1.298	0.870-1.938	0.201
Live birth rate	1.039	0.822-1.313	0.747	1.521	0.967-2.393	0.07
Miscarriage rate	0.925	0.595-1.437	0.729	0.880	0.417-1.857	0.738
Ectopic pregnancy	1.880	0.531-6.649	0.327	NA	NA	NA
Cesarean section	0.641	0.426-0.996	0.034^*^	1.751	0.594-5.156	0.310
preterm birth	0.810	0.487-1.347	0.416	2.444	0.105-5.670	0.578
Lowbirthweight	0.494	0.094-2.604	0.406	NA	NA	NA
Pregnancy-induced hypertension	0.274	0.049-1.532	0.140	0.531	0.998-1.007	0.693
Gestational Diabetes	0.468	0.111-1.970	0.300	0.038	0.002-0.707	0.028^*^

Adjusted for BMI, the number of abortion, endometrial preparation methods, endometrial thickness and pattern, the number of embryo transferred and embryos stage. OR, odds ratio; CI, confidence interval; NA:Due to an insufficient number of events, no meaningful statistical analysis was performed. *P < 0.05. This exploratory subgroup analysis was performed without adjustment for multiple comparisons and should therefore be interpreted with caution.

#### Women ≥35 years

The OI group had significantly higher IR, positive hCG rate, CPR, and LBR (*P* = 0.015), as well as a lower GDM rate (P = 0.032) compared to the HRT group. Adjusted analysis showed that OI was independently associated with a reduced GDM rate (OR = 0.038, 95% CI: 0.002–0.707; *P* = 0.028) and a trend toward a higher LBR (OR = 1.521, 95% CI: 0.967–2.393; *P* = 0.07) (**Tables 6**, **7**).

## Discussion

The primary finding of this study is that, after comprehensive adjustment for patient and cycle characteristics, the LE-HMG OI protocol and the standard HRT protocol resulted in comparable rates of both live birth and clinical pregnancy following FET. This conclusion regarding the primary outcomes aligns with the findings of Hosseini-Najarkolaei et al. from a randomized controlled trial employing a similar LE-HMG protocol (10). providing supportive evidence from a different study design for the equivalence of the two regimens in the overall population.

It is noteworthy that our conclusion appears to differ on the surface from several previous studies and meta-analyses that suggested superior outcomes with OI cycles (14–16). However, this apparent discrepancy primarily stems from differences in the level and focus of the conclusions. The advantages emphasized in references (14–16) were primarily established in specific populations with ovulatory disorders, such as PCOS. Our exploratory analysis further revealed that within the subgroup of women with ovulatory disorders, the LE-HMG combination was also associated with a higher CPR. This finding is not contradictory to the prior conclusions but rather extends them in two significant aspects: first, it validates the potential benefit of OI protocols for specific populations within a broader “ovulatory disorders” spectrum; second, it provides the first systematic evidence for the advantage of the LE-HMG combination protocol in this subgroup, offering clinicians an alternative to LE monotherapy that may have synergistic effects. The underlying mechanism may involve the combined action of LE and HMG in promoting follicular development, maintaining physiological feedback, and optimizing luteal support (17–19).

Of particular significance, this study represents, to our knowledge, the first systematic comparison of perinatal outcomes between this specific LE-HMG combination protocol and HRT in FET. Our exploratory analysis indicated that the LE-HMG OI protocol might be associated with a lower risk of cesarean section and GDM compared to the HRT cycle. A plausible mechanism for this potential advantage could be the presence of a functional corpus luteum in OI cycles. The corpus luteum secretes various vasoactive substances (e.g., relaxin, VEGF) believed to be crucial for early placentation and maternal cardiovascular adaptation to pregnancy (20, 21). In contrast, the fully exogenous hormone-controlled HRT cycle lacks this key physiological component, which might contribute to its different risk profile for obstetric complications (22). It is important to emphasize that these findings regarding perinatal outcomes are exploratory and require confirmation in prospective studies.

Exploratory analyses indicated that HRT was associated with a higher cesarean section rate not only in the overall population but also in subgroups with normal ovulation or aged <35 years. This observation might be partially explained by the trend toward a higher incidence of some obstetric complications (e.g., GDM, PIH) in HRT cycles, which could increase the likelihood of cesarean delivery (23–25). Meanwhile, our data suggested that OI cycles were associated with a lower GDM rate, an association particularly notable in the exploratory subgroup of women aged ≥ 35 years. If validated, this potential advantage could be meaningful for reducing long-term maternal and neonatal health risks.

Taken together, these exploratory findings suggest that the LE+HMG-based OI protocol may offer potential benefits beyond achieving live birth, including a possible reduction in obstetric complications. Practical considerations also appear favorable for OI, as its medication and luteal support are typically less expensive, easier to administer, and associated with fewer side effects than HRT. Therefore, if the potential reduction in obstetric complications such as GDM and cesarean section is confirmed in future studies, the OI protocol could represent a valuable strategy that balances efficacy, patient tolerability, and healthcare resource utilization.

### Limitations

This study has several limitations. First, as a single-center retrospective study, the assignment of endometrial preparation protocols was based on clinical judgment rather than randomization, introducing the potential for selection bias and confounding by indication. Although multivariate logistic regression was used to adjust for key confounders (e.g., BMI, endometrial parameters, embryo characteristics), and a homogeneity analysis was performed in **Supplementary Tables S1, S2**, the influence of residual confounding from unmeasured factors—such as detailed physician preference, patient compliance, or socioeconomic factors**—**cannot be entirely excluded. Second, the findings from a single institution may limit the generalizability to other populations or clinical settings.

It is crucial to emphasize that the subgroup analyses (stratified by ovulatory status and age) were exploratory in nature. These analyses were not adjusted for multiple comparisons. Furthermore, some subgroups (e.g., women aged ≥35 years) had relatively small sample sizes, resulting in wide confidence intervals (e.g., for GDM: OR = 0.038, 95% CI: 0.002–0.707). Therefore, these subgroup findings should be interpreted as hypothesis-generating exploratory evidence and require cautious interpretation and external validation.

Consequently, the primary conclusion of comparable LBRs between protocols, as well as the suggested potential benefits of the OI protocol regarding obstetric complications, needs to be confirmed by future prospective, multicenter randomized controlled trials. Future prospective, multicenter randomized controlled trials are needed to confirm these findings. Additionally, advanced analytical methods (e.g., propensity score matching) applied to larger retrospective datasets could help further mitigate confounding.

## Conclusions

In this retrospective cohort, LE-HMG OI and conventional HRT resulted in comparable LBRs after confounder adjustment. The OI protocol was also associated with a reduced risk of obstetric complications, specifically cesarean section and GDM. Exploratory subgroup analyses revealed potential variations in outcomes across different patient populations. These findings support the OI protocol as a valuable endometrial preparation strategy, with its potential perinatal benefits warranting further prospective validation.

## Data Availability

The raw data supporting the conclusions of this article will be made available by the authors, without undue reservation.
