# Supplementary material for: Reproductive and obstetric outcomes following frozen embryo transfer: letrozole combined with human menopausal gonadotropin versus hormone replacement cycle
Source: Front Endocrinol (Lausanne). 2026 Jan 29;17:1698208. doi: 10.3389/fendo.2026.1698208 (PMC12893984; doi:10.3389/fendo.2026.1698208)
Supplement: Supplementary file 1 [file Table1.docx]

**Supplementary Table 1.** Regression analysis of pregnancy and obstetric outcomes following cleavage-stage high-quality single embryo transfer.

| **outcomes** | **Odds ratio** | **95% confidence interval** | **P-value** |
| --- | --- | --- | --- |
| Clinical pregnancy rate | 0.771 | 0.497-1.196 | 0.245 |
| Live birth rate | 0.886 | 0.540-1.453 | 0.631 |
| Miscarriage rate | 1.022 | 0.413-2.529 | 0.963 |
| Ectopic pregnancy rate | 1.662 | 0.153-18.018 | 0.676 |
| Cesarean section | 0.349 | 0.134-0.908 | 0.031^*^ |
| preterm birth | 1.955 | 0.381-10.041 | 0.422 |
| Lowbirthweight | 3.165 | 0.310-32.348 | 0.331 |
| Pregnancy-induced hypertension | NA | NA | NA |
| Gestational Diabetes | 2.355 | 0.185-29.896 | 0.509 |

The reference group was the HRT group. An OR > 1 indicates a higher risk in the OI group, while an OR < 1 indicates a lower risk in the OI group. Adjusted for the women age, duration of infertility, history of abortion, BMI, AMH, infertility etiology, the endometrial thickness and type. OR, odds ratio; CI, confidence interval; NA：Due to an insufficient number of events, no meaningful statistical analysis was performed.

*P < 0.05.
