# Supplementary material for: Reproductive and obstetric outcomes following frozen embryo transfer: letrozole combined with human menopausal gonadotropin versus hormone replacement cycle
Source: Front Endocrinol (Lausanne). 2026 Jan 29;17:1698208. doi: 10.3389/fendo.2026.1698208 (PMC12893984; doi:10.3389/fendo.2026.1698208)
Supplement: Supplementary file 2 [file Table2.docx]

**Supplementary Table 2.** Regression analysis of pregnancy and obstetric outcomes following blastocyst-stage high-quality single embryo transfer.

| **outcomes** | **Odds ratio** | **95% confidence interval** | **P-value** |
| --- | --- | --- | --- |
| Clinical pregnancy rate | 1.150 | 0.821-1.611 | 0.417 |
| Live birth rate | 0.954 | 0.677-1.344 | 0.787 |
| Miscarriage rate | 0.823 | 0.431-1.574 | 0.556 |
| Ectopic pregnancy rate | 1.052 | 0.084-13.240 | 0.969 |
| Cesarean section | 0.431 | 0.231-0.823 | 0.010^*^ |
| preterm birth | 0.664 | 0.300-1.470 | 0.313 |
| Lowbirthweight | 1.632 | 0.450-5.920 | 0.456 |
| Pregnancy-induced hypertension | NA | NA | NA |
| Gestational Diabetes | 7.478 | 0.679-82.343 | 0.100 |

The reference group was the HRT group. An OR > 1 indicates a higher risk in the OI group, while an OR < 1 indicates a lower risk in the OI group. Adjusted for the women age, duration of infertility, history of abortion, BMI, AMH, infertility etiology, the endometrial thickness and type. OR, odds ratio; CI, confidence interval; NA：Due to an insufficient number of events, no meaningful statistical analysis was performed.

*P < 0.05.
